# Malignant Transformation of an Odontogenic Cyst in a Period of 10 Years

**DOI:** 10.1155/2014/762969

**Published:** 2014-04-07

**Authors:** Juliane Pirágine Araújo, Luiz Paulo Kowalski, Mônica Lúcia Rodrigues, Oslei Paes de Almeida, Clovis Antonio Lopes Pinto, Fabio Abreu Alves

**Affiliations:** ^1^Stomatology Department, School of Dentistry, São Paulo University, São Paulo, SP, Brazil; ^2^Head and Neck Surgery and Otorhinolaryngology Department, AC Camargo Cancer Center, São Paulo, SP, Brazil; ^3^Department of Oral Diagnosis, Piracicaba Dental School, University of Campinas (UNICAMP), Piracicaba, SP, Brazil; ^4^Pathology Department, AC Camargo Cancer Center, São Paulo, SP, Brazil; ^5^Stomatology Department, AC Camargo Cancer Center, Rua Professor Antônio Prudente, 211 Liberdade, 01509-010 São Paulo, SP, Brazil

## Abstract

Primary intraosseous carcinoma of the jaws (PIOSCC) might arise from odontogenic epithelium, more commonly from a previous odontogenic cyst. The aim of this case is to illustrate that the clinician should consider that an apparent benign dentigerous cyst can suffer malignant transformation and that all material removed from a patient must be evaluated histologically. A 44-year-old man presented in a routine periapical X-ray an impacted lower left third molar with radiolucency over its crown. Ten years later, the patient complained of pain in the same region and the tooth was extracted. After one month, the patient still complained of pain and suffered a fracture of the mandible. A biopsy was performed and carcinoma was diagnosed. The patient was treated surgically with adjuvant radio- and chemotherapy and after 8 years, he is well without signs of recurrences. This report describes a central mandibular carcinoma probably developed from a previous dentigerous cyst.

## 1. Introduction

Primary intraosseous squamous cell carcinomas of the jaws (PIOSCC) are aggressive malignancies mainly derived from odontogenic epithelium [1]. These carcinomas can be aggressive, involving large areas of the jaws, but the features are usually nonspecific and biopsy confirms the diagnosis [2, 3]. The two-to-six-year survival rate is approximately 53% and local recurrence has been the major problem in patients not treated with radical excision [1].

Malignant transformation of odontogenic cysts is estimated to be between 0.13% and 2%, with most of the cases involving the mandible [3]. In addition, the aim of this report was to describe a case of an intraosseous oral squamous cell carcinoma, probably derived from an odontogenic cyst.

## 2. Case Report

**Figure 1:**
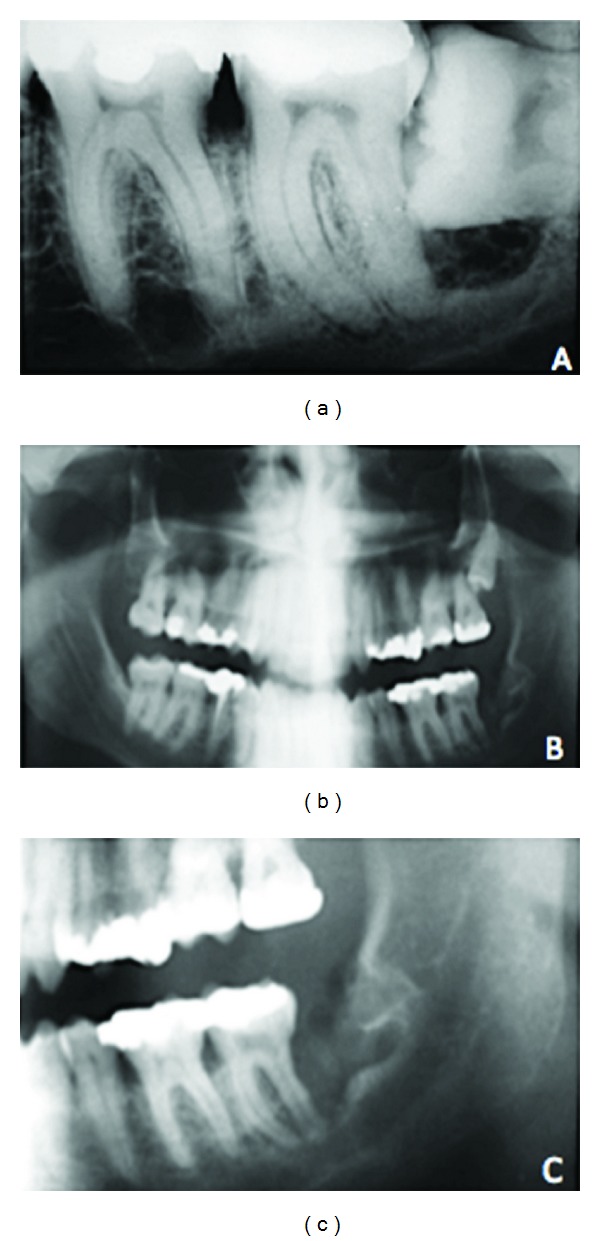
(a) Periapical X-ray showing a radiolucency surrounding the crown of the left third molar suggestive of dentigerous cyst. (b-c) Panoramic X-ray performed after 15 days of the third molar extraction showing no suggestive malignant transformation.

A 44-year-old man was referred to our cancer center for treatment of a jaw tumor. The medical history revealed that in 1994 the patient underwent a periapical X-ray of an impacted lower left third molar, showing radiolucency over the crown interpreted as dentigerous cyst (Figure 1(a)). There were no symptoms and the patient and his dentist decided just to follow up. After 11 years the patient presented pain in the region and both tooth and lesion were removed and discarded. After 15 days (Figures 1(b) and 1(c)), the patient continued to present pain and a panoramic X-ray showed a local tooth extraction with no signs of malignant lesion. A biopsy was performed and the diagnosis was of squamous cell carcinoma (SCC).

**Figure 2:**
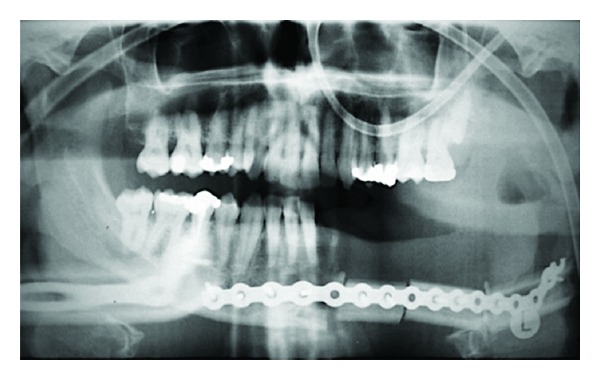
Immediately postsurgical treatment panoramic X-ray, which shows fibula free flap reconstruction of the mandible.

**Figure 3:**
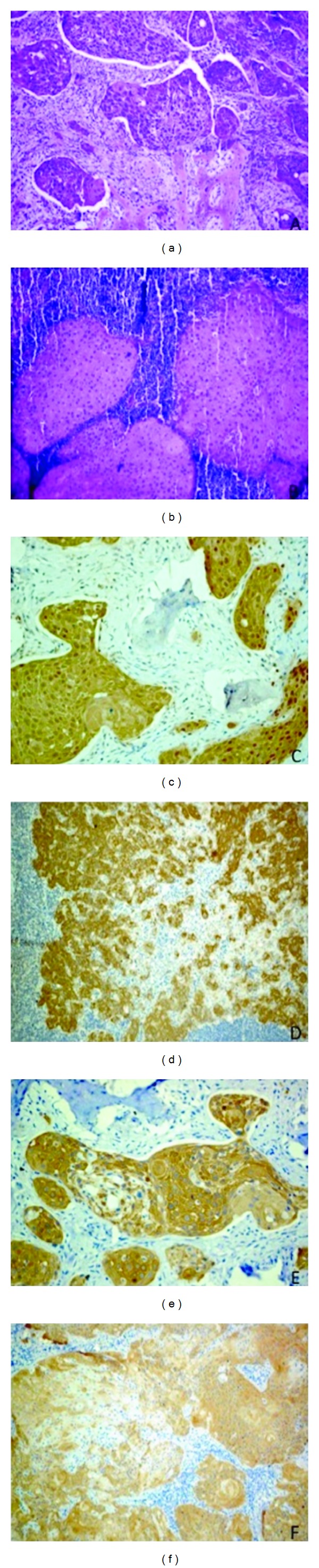
(a) Squamous cell carcinoma islands infiltrating the bone trabeculae. (b) Regional metastasis (HE 200x). (c) CK 5 immunohistochemical expression in the primary tumor. (d) CK 5 in regional metastasis. (e) CK 14 expression in the primary tumor. (f) CK 14 in the regional metastasis.

Treatment consisted of partial mandibulectomy with disarticulation and supraomohyoid neck dissection of the left side followed by microsurgical reconstruction with fibula free flap (Figure 2). The histopathological analysis confirmed an intraosseous SCC with regional metastasis in 1 (level Ib) out of 36 lymph nodes, without capsular rupture (Figures 3(a) and 3(b)). An immunohistochemical analysis with cytokeratins 5 and 14 was performed in the primary tumor and in a regional lymph node and they were positive in both sites (Figures 3(c)–3(f))

**Figure 4:**
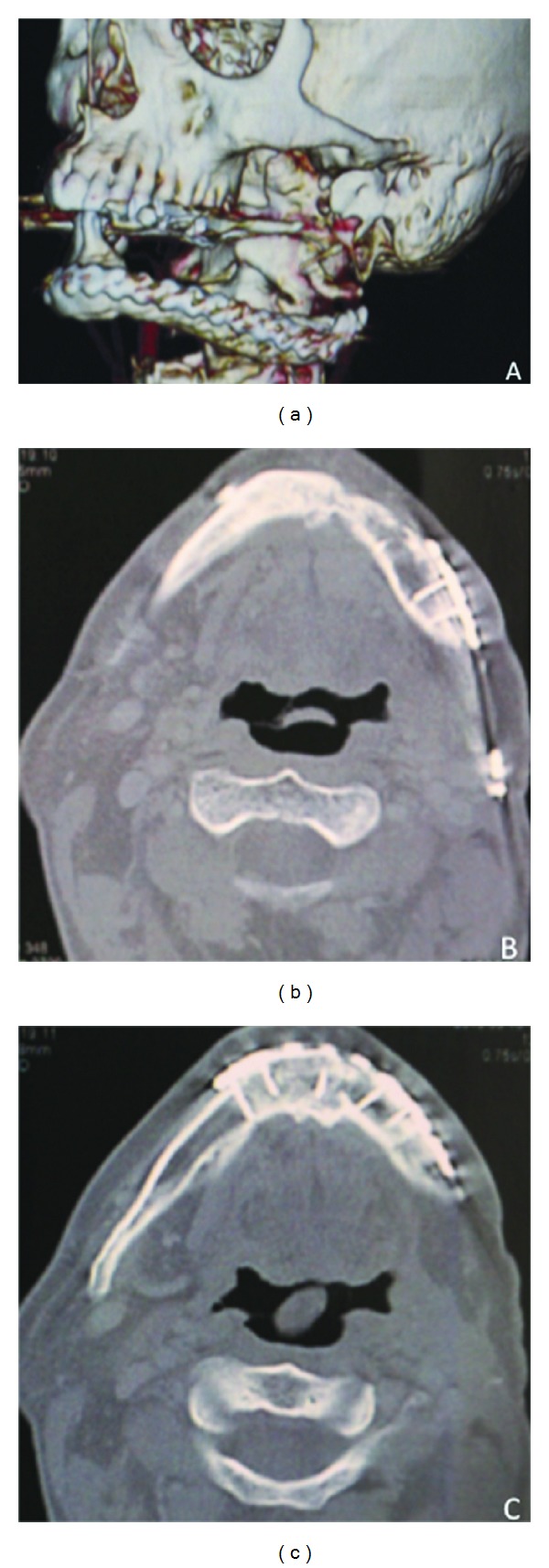
Computed tomography (CT) performed after 8 years of followup. (a) Three-dimensional reconstruction illustrates the osseous consolidation between the mandible and the fibula. (b-c) Axial slices CT showing no evidence of local recurrence.

The adjuvant treatment consisted of radiotherapy (total radiation dose of 64.4 Gy) associated with cycles of cisplatin (100 mg/m^2^ every 21 days). The patient has been followed for 8 years without evidence of recurrences (Figure 4).

## 3. Discussion

PIOSCC is rare and for a correct diagnosis the possibilities of a SCC of the oral mucosa, other types of odontogenic carcinomas, and metastasis must be excluded. The present case clearly shows an intraosseous lesion consistent with dentigerous cyst that was diagnosed as malignant tumor after 11 years. Therefore, it is reasonable to consider that the final diagnosis is of a PIOSCC derived from dentigerous cyst.

Most cases of PIOSCC involve the mandible, causing swelling, pain, and paresthesia of the lower lip [4, 5]. The chief complain on the present case was pain which occurred only after 11 years of detection of a suggestive image of dentigerous cyst in a periapical X-ray. Radiographically PIOSCC may present as unilocular or multilocular lesions, with ill-defined or well-defined but uncorticated borders [5]. Our case showed initially as unilocular well-defined lesion very suggestive of dentigerous cyst and after 11 years when the tooth was removed there was no significant alteration which could suggest a malignant transformation.

It is well accepted that odontogenic epithelium of cysts and benign tumours may suffer malignant transformation, including also rests of Malassez and of the dental lamina [6, 7]. Nevertheless, the mechanisms involved are not known, and as these cases are rare and usually not very well documented, many aspects are still controversial. Whenever possible, it is important to find in the same lesion areas of carcinoma adjacent to the benign epithelium from which the malignancy derived. Unfortunately, the removed tooth and its associated tissues were not sent for histopathological examination, and this is still not uncommon in most parts of the world.

Various odontogenic cysts have been associated with PIOSCC, including residual cyst, dentigerous cyst, odontogenic keratocyst, calcifying odontogenic cyst, and lateral periodontal cyst [5]. In analysis of 116 reported cases of PIOSCC arising in odontogenic cysts, the type of cyst more observed was residual/radicular with 70 cases followed by dentigerous cyst with 19 cases, keratocystic odontogenic tumor with 16 cases, 1 case of lateral periodontal, and 9 unclassified cases [8]. The identification of the benign epithelial lining of a preexisting odontogenic cyst characterizes PIOSCC type 1 (ex odontogenic cyst); unfortunately we failed in demonstrating type 1 PIOSCC in our case because the material was discarded. Consequently, the tumor was better classified as type 3 PIOSCC (arising de novo).

Expression of cytokeratins (CK) in developing tooth germ can be helpful to understand the histogenesis of either odontogenic cysts or benign and malignant tumors. CK 5, 7, 8, 14, and 19 are expressed in the enamel organ [9, 10] and CK 14 is the main intermediate filament found in the dental lamina, reduced enamel epithelium, and enamel organ; nevertheless, this CK also is commonly expressed in other epithelia including the oral mucosa [11]. With the objective of confirming the possible odontogenic origin of our case, CK 5 and CK 14 were performed, and both CK were strongly immunoreactive in primary and metastatic tumors. These findings may enhance the hypothesis of malignant transformation of the dentigerous cyst.

Metastasis to cervical lymph nodes is observed in up to 50% of all cases of PIOSCC, and it can also spread along the inferior alveolar nerve, usually requiring an aggressive treatment to control the disease [8, 12, 13]. Adjuvant radiotherapy may be indicated on dependence of tumor extension and regional node involvement. The present case showed a cervical lymph node involvement. However, mortality appears to be more related to local extension of the neoplasm than metastatic disease [3, 12, 14–16]. According to Thomas et al. (2000) survival rates of 1, 2, and 3 years were 75.7%, 62.1%, and 37.8%, respectively, indicating the poor prognosis and necessity of an adequate treatment. Our patient underwent a left hemimandibulectomy with left supraomohyoid neck dissection followed by microsurgical reconstruction with fibula free flap and adjuvant radiotherapy associated with chemotherapy. The patient has been followed for 8 years without any evidence of recurrences. In summary, we report a case of a probable dentigerous cyst that after 11 years showed malignant transformation, illustrating that, despite most odontogenic lesions being benign, all clinicians must be aware of the possibility of odontogenic malignancies. PIOSCC commonly causes pain involving large areas of the jaws, but eventually the early diagnosis may be a challenge as the clinical and imaginological features can be unspecific.

This case also illustrates that a careful examination and regular followup of patients with impacted teeth with an associated radiolucency are important. In addition, histopathological examination of odontogenic cysts or pericoronal tissue is recommended to confirm the clinical diagnosis and due to its possibility of malignant transformation.
